# Whole‐body non‐forensic fetal virtopsy using postmortem magnetic resonance imaging at 7 Tesla *vs* classical autopsy

**DOI:** 10.1002/uog.29106

**Published:** 2024-10-07

**Authors:** A. Staicu, C. Albu, R. Popa‐Stanila, C. Bondor, L. Chiriac, D. Eniu, I. Goidescu, A. R. Florian, M. Surcel, G. Cruciat, D. Muresan, I. Rotar

**Affiliations:** ^1^ Mother and Child Department, 1^st^ Clinic of Obstetrics and Gynaecology ‘Iuliu Haţieganu’ University of Medicine and Pharmacy Cluj‐Napoca Romania; ^2^ Department of Pathology ‘Iuliu Haţieganu’ University of Medicine and Pharmacy Cluj‐Napoca Romania; ^3^ Centre of Advanced Research Studies Emergency County Hospital, IMOGEN Cluj‐Napoca Romania; ^4^ Department of Radiology ‘Iuliu Haţieganu’ University of Medicine and Pharmacy Cluj‐Napoca Romania; ^5^ Department of Medical Informatics and Biostatistics ‘Iuliu Haţieganu’ University of Medicine and Pharmacy Cluj‐Napoca Romania; ^6^ Department of Medical Biophysics ‘Iuliu Haţieganu’ University of Medicine and Pharmacy Cluj‐Napoca Romania; ^7^ National Magnetic Resonance Centre Babeș Bolyai University Cluj‐Napoca Romania; ^8^ Faculty of Physics Babeș Bolyai University Cluj‐Napoca Romania

**Keywords:** fetal autopsy, high‐field magnetic resonance imaging, MRI, postmortem magnetic resonance imaging, postmortem whole‐body fetal imaging, virtopsy, virtual autopsy

## Abstract

**Objective:**

To determine the diagnostic accuracy of virtual autopsy using whole‐body postmortem ultra‐high field magnetic resonance imaging (MRI) at 7 Tesla (T), using a short T2‐weighted imaging (T2‐WI) protocol, compared with classical autopsy, for detecting structural abnormalities in small second‐trimester fetuses.

**Methods:**

Thirty consecutive fetuses at 13–19 weeks' gestation (weight, 17–364 g) were included following spontaneous pregnancy loss or termination of pregnancy. After fixation in 10% formaldehyde solution (48 h to 1 week), all fetuses were scanned using a two‐dimensional turbo high‐resolution T2‐WI protocol with multislice relaxation time, followed by an invasive autopsy. The diagnostic accuracy of virtual autopsy *vs* classical autopsy was calculated for 990 anatomical structures (30 fetuses × 33 items). Sensitivity, specificity, positive and negative predictive values and Cohen's κ coefficient of agreement, with their 95% CIs, as well as the McNemar test, were used to evaluate the accuracy and agreement of the two diagnostic methods. Analysis was stratified by anatomical segment (nervous, pulmonary, cardiovascular, digestive, renal, facial and skeletal) and across three gestational‐age intervals (13–14, 15–16 and 17–19 weeks).

**Results:**

Considering classical autopsy as the gold standard, virtual autopsy had a sensitivity of 92.04% (95% CI, 85.42–96.29%) and a specificity of 97.87% (95% CI, 94.64–99.42%), with a positive predictive value of 96.30% (95% CI, 90.78–98.56%) and a negative predictive value of 95.34% (95% CI, 91.61–97.45%), achieving a diagnostic accuracy of 95.68% (95% CI, 92.73–97.68%) for detecting structural abnormalities in second‐trimester fetuses. Cohen's κ for virtual *vs* classical autopsy was 0.907. The diagnostic ability of virtual autopsy at 7 T for malformed fetuses was superior to that of classical autopsy for analyzing the nervous system in small fetuses with pronounced autolysis, equivalent to that of classical autopsy when analyzing pulmonary, cardiovascular and renal systems and inferior when evaluating the fetal intestines. The sensitivity of virtual autopsy at 7 T for describing structural abnormalities increased with gestational age.

**Conclusion:**

Virtual fetal autopsy using 7‐T MRI and a turbo high‐resolution T2‐WI protocol with multislice relaxation time is a feasible postmortem diagnostic tool for the identification of fetal structural anomalies. © 2024 The Author(s). *Ultrasound in Obstetrics & Gynecology* published by John Wiley & Sons Ltd on behalf of International Society of Ultrasound in Obstetrics and Gynecology.

## Abstract 2

This article's abstract has been translated into Spanish and Chinese. Follow the links from the abstract to view the translations.

## RESUMEN

**Autopsia virtual fetal no forense de cuerpo entero mediante resonancia magnética póstuma a 7 Tesla *versus* autopsia clásica**

**Objetivo:**

Determinar la precisión diagnóstica de la autopsia virtual póstuma de cuerpo entero mediante resonancia magnética (RM) de campo ultra alto a 7 Tesla (7T), utilizando un protocolo corto de imágenes ponderadas en T2 (T2‐WI), en comparación con la autopsia clásica, para detectar anomalías estructurales en fetos pequeños del segundo trimestre.

**Métodos:**

Se incluyeron 30 fetos consecutivos de 13–19 semanas de gestación (peso 17–364 g) tras una pérdida espontánea del embarazo o una interrupción del embarazo. Tras la fijación en solución de formaldehído al 10% (de 48 h a 1 semana), todos los fetos fueron examinados mediante un protocolo de T2‐WI bidimensional turbo de alta resolución con tiempo de relajación multicorte, después de una autopsia clásica. Se calculó la precisión diagnóstica de la autopsia virtual frente a la de la autopsia clásica para 990 estructuras anatómicas (30 fetos×33 elementos). Se utilizaron la sensibilidad, la especificidad, los valores predictivos positivo y negativo y el coeficiente de concordancia κ de Cohen, con sus IC 95%, así como la prueba de McNemar, para evaluar la precisión y la concordancia de los dos métodos de diagnóstico. El análisis se estratificó por segmentos anatómicos (nervioso, pulmonar, cardiovascular, digestivo, renal, facial y esquelético) y para tres intervalos de edad gestacional (13–14, 15–16 y 17–19 semanas).

**Resultados:**

Considerando la autopsia clásica como el patrón de referencia, la autopsia virtual tuvo una sensibilidad del 92,04% (IC 95%, 85,42–96,29%) y una especificidad del 97,87% (IC 95%, 94,64–99,42%), con un valor predictivo positivo del 96,30% (IC 95%, 90,78–98,56%) y un valor predictivo negativo del 95,34% (IC 95%, 91,61–97,45%), lo que supuso una precisión diagnóstica del 95,68% (IC 95%, 92,73–97,68%) para detectar anomalías estructurales en fetos del segundo trimestre. La κ de Cohen para la autopsia virtual frente a la clásica fue de 0,907. La capacidad diagnóstica de la autopsia virtual en 7T para fetos malformados fue superior a la de la autopsia clásica para el análisis del sistema nervioso en fetos pequeños con autólisis pronunciada, equivalente a la de la autopsia clásica al analizar los sistemas pulmonar, cardiovascular y renal e inferior al evaluar los intestinos fetales. La sensibilidad de la autopsia virtual en 7T para describir anomalías estructurales aumentó con la edad gestacional.

**Conclusión:**

La autopsia fetal virtual utilizando la RM 7T y un protocolo turbo de alta resolución T2‐WI con tiempo de relajación multicorte es una herramienta de diagnóstico póstumo viable para la identificación de anomalías estructurales fetales.

## 摘要

**利用7特斯拉死后磁共振成像进行全身非法医胎儿虚拟尸检 与传统尸检的比较**

**目的:**

通过采用短 T2 加权成像(T2‐WI)方案，使用 7 特斯拉(T)全身死后超高场磁共振成像(MRI)进行虚拟尸检，与传统尸检进行比较，确定虚拟尸检在检测孕中期小胎儿结构异常方面的诊断准确性。

**方法:**

连续纳入 30 个妊娠 13‐19 周(体重 17‐364 克)的自然流产或终止妊娠胎儿。所有胎儿在 10% 甲醛溶液中固定(48 小时至 1 周)后，使用多层弛豫时间二维涡轮高分辨率 T2‐WI 方案进行扫描，然后进行有创尸检。对 990 个解剖结构(30 个胎儿×33 个项目)计算了虚拟尸检与传统尸检的诊断准确性。使用灵敏度、特异性、阳性和阴性预测值、科恩卡帕一致性系数及其 95%CI 以及麦克尼马尔检验评估两种诊断方法的准确性和一致性。分析按解剖部位(神经、肺、心血管、消化、肾、面部和骨骼)和三个孕龄间隔(13‐14 周、15‐16 周和 17‐19 周)进行分层。

**结果:**

将传统尸检作为金标准，虚拟尸检的敏感性为 92.04%(95% CI，85.42‐96.29%)，特异性为 97.87%(95% CI，94.64‐99.42%)，阳性预测值为 96. 30%(95% CI，90.78‐98.56%)，阴性预测值为 95.34%(95% CI，91.61‐97.45%)，检测孕中期胎儿结构异常的诊断准确率为 95.68%(95% CI，92.73‐97.68%)。虚拟尸检与传统尸检的科恩卡帕系数为 0.907。7T 虚拟尸检对畸形胎儿的诊断能力优于传统尸检，可分析有明显自溶的小胎儿的神经系统，与传统尸检分析肺、心血管和肾脏系统的诊断能力相当，而评估胎儿肠道的诊断能力较差。7T 虚拟解剖对结构异常的敏感度随胎龄的增加而增加。

**结论:**

利用 7T 磁共振成像和具有多层弛豫时间的涡轮高分辨率 T2‐WI 方案进行虚拟胎儿尸检，是鉴定胎儿结构异常的可行尸检诊断工具。

**CONTRIBUTION**

*What are the novel findings of this work?*

Virtual fetal autopsy using whole‐body postmortem ultra‐high field (UHF) magnetic resonance imaging (MRI) at 7 Tesla (T), with 10% formaldehyde fixation and a T2‐weighted imaging protocol, is a feasible diagnostic tool for the identification of structural anomalies of the nervous, pulmonary, cardiovascular and renal systems in small second‐trimester fetuses. The diagnostic sensitivity increases with gestational age.

*What are the clinical implications of this work?*

Considering the high pathologist workload in tertiary obstetric centers, virtual fetal autopsy using UHF‐MRI at 7 T could help to triage cases of fetal demise that would benefit from complex dissection procedures. Virtual autopsy limits human contact while offering high‐quality information for diagnosis.

## INTRODUCTION

To accommodate the parental need for closure after fetal/neonatal death, while acknowledging the fear of disfigurement by autopsy felt by some parents^1^, perinatal postmortem imaging (virtual autopsy or ‘virtopsy’) aims to provide information on the cause of death in a less invasive manner compared with classical autopsy^2^. The most apparent disadvantage of virtopsy is the lack of histological samples^3^. After analyzing more than 5000 pediatric autopsies, recent work showed that, if antenatal ultrasonography and postmortem magnetic resonance imaging (MRI) findings align, conventional autopsy provides valuable extra information in less than 5% of cases^2^, ^4^.

Postmortem imaging could be a useful triage method for selecting cases that may genuinely benefit from complex classical autopsy, which requires time, specific materials and dedicated personnel. Ultra‐high magnetic field (UHF)‐MRI with a strength of 7–11.7 Tesla (T) has been used to provide detailed images of the fetal anatomy for fetal virtopsy^5^. However, 9‐T and 11‐T scanners are intended only for research purposes, and their utility in daily clinical practice is limited^6^, ^7^. The most recent review of postmortem imaging techniques found that fewer than 20 studies in the last 28 years have focused on postmortem fetal imaging using UHF‐MRI^8^. Only three studies compared whole‐body postmortem UHF‐MRI with fetal autopsy^6^, ^7^, ^9^. UHF‐MRI at 7 T may represent the most promising modality for clinical application because the US Food and Drug Administration has already approved for clinical use a 7‐T device (Magnetom Terra; Siemens Medical Solutions Inc., Malvern, PA, USA).

Current research on UHF‐MRI is limited by the coil gantry, which only allows for the examination of fetuses weighing less than 300 g; however, clinical 7‐T scanners allow acquisition of the entire fetal body, regardless of size, in one volume, reducing the scanning time. One important step before introducing fetal virtopsy at 7 T into clinical practice is to demonstrate its feasibility and establish a suitable scanning protocol.

The aim of this study was to assess the diagnostic usefulness of virtual autopsy of small second‐trimester fetuses using UHF‐MRI at 7 T, with classical autopsy as the reference standard. We used a short, simple T2‐weighted imaging (T2‐WI) scanning protocol that can be easily reproduced to examine fetuses weighing less than 300 g. We also investigated the influence of fetal weight and gestational age on diagnostic accuracy.

## METHODS

This was a prospective longitudinal study conducted between March 2016 and January 2019 in the 1st Clinic of Obstetrics and Gynecology, Emergency County Clinical Hospital (ECCHCN), Cluj‐Napoca, Romania. The Standards for Reporting Qualitative Research checklist was used to prepare this report^10^. The Ethics Committee of the ‘Iuliu Haţieganu’ University of Medicine and Pharmacy approved the protocol (No. DEP50/22.02.2022). Before inclusion, women signed an informed consent form, in accordance with the World Medical Association Declaration of Helsinki (2000).

Thirty consecutive singleton fetuses at 13–19 weeks' gestation, weighing 17–364 g, were enrolled following spontaneous pregnancy loss or termination of pregnancy. Indications for termination were karyotype anomaly or severe malformation syndrome. In the case of spontaneous pregnancy loss, the intrauterine retention period was assessed by subtracting from the gestational age at delivery (calculated from the date of the last menstrual cycle) the approximate gestational age estimated from femur length (measured by prenatal ultrasonography).

Termination of pregnancy was carried out in accordance with the local legal framework using 200 mg of oral mifepristone followed by transvaginal administration of 800 mg of prostaglandins after 48 h, according to internal departmental protocol. After delivery, the fetus and placenta were transported to IMOGEN, the Medical Research Institute within ECCHCN, in compliance with the Human Tissue Act (2004). The fetus was soaked in 10% formaldehyde solution at room temperature (20–23°C) for between 48 h and 1 week, and subsequently stored at 4°C until scanning. Every fetus was subjected to two morphological analyses: first by MRI using a 7‐T Bruker Biospec 70/16 UltraShielded and Refrigerated machine (Bruker BioSpin MRI GmbH, Ettlingen, Germany) with a high magnetic field gradient unit (BGA‐9‐SHP) and later by stereomicroscopic fetal autopsy according to the current protocol of the fetal pathology department. All information was centralized in a database by a statistician without medical knowledge.

### Postmortem 7‐T MRI examination

During the postmortem MRI examination, the fetus was placed in an adjustable plastic bag (to avoid corrosion of the probe) without formaldehyde solution and scanned in the supine position, head first. Images of the head and neck, thorax, abdomen and pelvis were acquired in axial, sagittal and coronal sections.

Prior to interpretation, the images were processed using a ParaVision 5.1 workstation (Bruker). A two‐dimensional tripilot protocol was used with a field‐of‐view of 4 cm and a slice thickness of 1.5 mm, with acquisition of 3 × 5 images in the axial, sagittal and coronal planes. The repetition time was 80 ms, the echo time was 1.8 ms and the chosen matrix was 256 × 256, with a resolution for this image size of 0.0234 cm/pixel.

A 60‐mm RES 300‐MHz 1H 089/060 QUAD TR radiofrequency volume coil (Bruker) was used for the two‐dimensional T2‐WI turbo high‐resolution protocol with multislice relaxation time. The acquisition parameters were modified to achieve a resolution between 0.0102 and 0.0104 cm/pixel, with image sizes between 2 cm and 5 cm, a matrix of 384 × 384 and a bandwidth equal to 62 500 Hz. The number of repetitions for scan enhancement was five at a temperature of 20°C for 0.5‐mm slices with an interslice distance of 0.75 mm. The repetition time, adjusted according to the number of acquired slices, varied between 3442 ms and 62 909 ms, depending on their size at an echo time of 36 ms. Details regarding the modified acquisition parameters according to fetal characteristics are provided in Table S1.

### Image analysis

Images were analyzed separately by a radiologist (R.P.‐S.) with expertise in fetal and pediatric radiology and an embryologist (A.S.) with experience in postmortem imaging, who were blinded to the clinical and prenatal investigations and the results of classical autopsy, using a standardized protocol. Interobserver variability was assessed by calculating the overall intraclass correlation coefficient (ICC) and its 95% CI using a two‐way mixed model with absolute agreement of single measurements.

An Advantage Workstation 4.6 (GE Healthcare, Chicago, IL, USA) was used to process images. Thirty‐three items from seven anatomical segments commonly assessed during conventional autopsy (nervous, pulmonary, cardiovascular, digestive and renal systems, facial region and skeleton) were analyzed for each case. The evaluated anatomical structures are listed in Table 1.

**Table 1:** Diagnostic utility of virtual autopsy using magnetic resonance imaging at 7 Tesla *vs* classical autopsy for detecting structural abnormalities in fetal anatomical segments evaluated according to a standard protocol

Anatomical segment*	Sensitivity (%)	Specificity (%)	PPV (%)	NPV (%)	Accuracy (%)	Cohen's κ	Malformation frequency (%)	*P* †	Structures evaluated
Nervous system (*n* = 210)	100 (79.41–100)	88.24 (63.56–98.54)	88.89 (68.52–96.71)	100 (100–100)	93.94 (79.77–99.26)	0.8790	48.48 (30.80–66.46)	0.157	Cerebral hemispheres, ventricular system, thalamus, hypophysis, corpus callosum, cerebellum, inner ear
Pulmonary system (*n* = 90)	100 (100–100)	96.30 (81.03–99.91)	75.00 (19.41–99.37)	100 (100–100)	96.67 (82.78–99.92)	0.8387	10.00 (01.11–26.53)	0.317	Trachea, bronchi, lungs
Cardiovascular system (*n* = 180)	84.62 (54.50–98.08)	94.12 (71.31–99.85)	91.67 (61.83–98.68)	88.89 (68.98–96.64)	88.89 (73.94–96.89)	0.7945	43.33 (25.46–62.57)	0.456	Four‐chamber image, atrioventricular valves, three‐vessel cross‐section, aorta, pulmonary artery, veins (superior and inferior vena cava)
Digestive system (*n* = 240)	73.33 (44.90–92.21)	100 (83.89–100)	100 (100–100)	84.00 (69.40–92.40)	88.89 (73.94–96.89)	0.7623	41.67 (25.51–59.21)	0.668	Appearance of diaphragm, integrity of abdominal wall, esophagus, stomach, liver, spleen, pancreas, bowels
Renal system (*n* = 90)	100 (100–100)	95.45 (77.16–99.88)	90.00 (57.01–98.39)	100 (100–100)	96.77 (83.30–99.92)	0.9242	29.03 (14.22–48.04)	0.500	Kidneys, bladder, genitals
Facial region (*n* = 90)	100 (100–100)	100 (100–100)	100 (100–100)	100 (100–100)	100 (100–100)	1.0000	70.45 (44.93–92.01)	< 0.001	Palate, mandible, orbits
Skeleton (*n* = 90)	71.43 (29.04–96.33)	100 (85.18–100)	100 (100–100)	92.00 (78.09–97.38)	93.33 (77.93–99.18)	0.7851	23.33 (9.93–42.28)	0.240	Anomalies of closure and shape of spinal column, anomalies of closure and shape of skull, presence or absence and anomalies of limbs

Data in parentheses are 95% CI.

* *n* values indicate total number of structures evaluated: 30 fetuses × number of structures evaluated per segment.

† McNemar test.

NPV, negative predictive value; PPV, positive predictive value.

For each case, the total number of structural malformations detected per anatomical segment analyzed was recorded, and a final diagnosis, including all anomalies, was established at the end of the examination. Images relevant to the diagnosis were attached to the individual files.

### Postmortem pathological examination

Pathological examination of all fetuses was performed blinded to the results of virtual autopsy by a pathologist (C.A.) specializing in fetal pathology according to the autopsy protocol of the French Society of Fetal Pathology^11^ in the pathology department at IMOGEN. Details of the equipment and techniques for pathological examination have been described previously^6^.

### Statistical analysis

SPSS version 15.0 (SPSS Inc., Chicago, IL, USA), Statistica version 8.0 (StatSoft Inc., Tulsa, OK, USA) and Microsoft Excel 2016 (Microsoft, Redmond, WA, USA) were used for statistical analysis. *P* < 0.05 was considered to indicate statistical significance. To evaluate the diagnostic utility of fetal high‐field virtopsy compared with stereomicroscopic conventional autopsy, the sensitivity, specificity and positive and negative predictive values, with their 95% CIs, were calculated using the online Interactive Statistics page^12^. The agreement between the two diagnostic methods was assessed using Cohen's κ coefficient with 95% CI, as well as the McNemar test.

We calculated the sample size using a methodology that considers the expected sensitivity and specificity in conditions of specific prevalence and Type‐I and Type‐II error rates^13^. The sample size was computed for the nervous segment, facial region, cardiovascular, pulmonary and renal systems and skeleton, based on our previous work^6^. We considered a 95% confidence level and precision of 0.1. The necessary sample size was calculated to be a minimum of 26 cases^14^, but this sample size calculation did not consider the digestive system.

## RESULTS

The diagnostic accuracy of virtual autopsy using MRI at 7 T *vs* classical autopsy was calculated based on the evaluation of 990 anatomical structures (30 fetuses × 33 items). Classical autopsy, which was considered the gold standard, identified 371 structural anomalies, representing a rate of 37.47% (95% CI, 32.05–43.28%). For virtual autopsy, the average scan time per case was 61.89 min and varied according to fetal weight, ranging from 49.28 min for a 21‐g fetus to 315 min for a 300‐g fetus. Agreement between the experts interpreting the postmortem images was very good, with an ICC of 0.984 (95% CI, 0.982–0.986). The baseline characteristics of the study population are summarized in Table 2.

**Table 2:** Baseline characteristics of study population (*n* = 30)

Characteristic	Value
Maternal age (years)	30.5 (17–39)
Gestational age (weeks)	15.8 (13–19)
Fetal weight (g)	119.4 (17–364)
Fetal sex	
Female	12 (40.0)
Male	17 (56.7)
Undifferentiated genitalia	1 (3.3)
Genetic testing	
Normal	10 (33.3)
Abnormal*	10 (33.3)
No genetic testing	10 (33.3)
Cause of fetal demise	
Termination of pregnancy	25 (83.3)
Spontaneous miscarriage	5 (16.7)

Data are given as mean (range) or *n* (%).

* Abnormal karyotype, trisomy 21, trisomy 18, triploidy.

### Overall diagnostic accuracy of fetal virtopsy

Using classical autopsy as the gold standard, virtual autopsy demonstrated a sensitivity of 92.04% (95% CI, 85.42–96.29%) and a specificity of 97.87% (95% CI, 94.64–99.42%), with a positive predictive value of 96.30% (95% CI, 90.78–98.56%) and a negative predictive value of 95.34% (95% CI, 91.61–97.45%), achieving a diagnostic accuracy for detecting structural abnormalities in second‐trimester fetuses of 95.68% (95% CI, 92.73–97.68%). Cohen's κ was 0.907, indicating an almost perfect agreement between the two methods. The concordance between classical and virtual autopsy was confirmed by the result of the McNemar test (*P* = 0.212), indicating no statistically significant difference between the two methods.

### Diagnostic accuracy of fetal virtopsy according to anatomical segment

The diagnostic accuracy of virtual autopsy compared with that of classical autopsy for specific anatomical segments is shown in Table 1. 7‐T UHF‐MRI displayed excellent overall sensitivity and specificity for each anatomical structure considered in all analyzed segments, except for the digestive system and skeleton.

**Figure 1:**
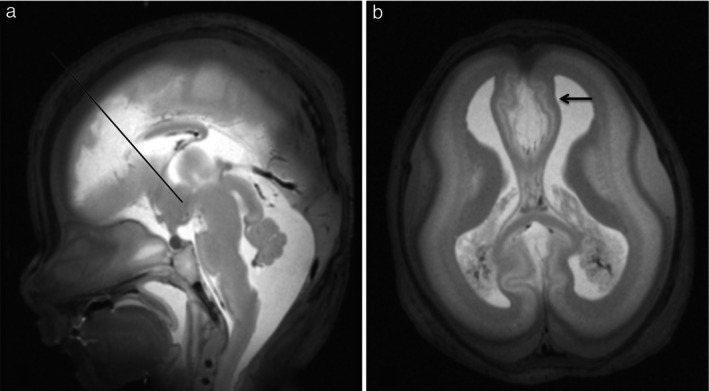
Fetal virtopsy using 7‐Tesla turbo spin‐echo high‐resolution T2‐weighted magnetic resonance imaging in fetus at 16 weeks' gestation following termination of pregnancy for early oligohydramnios and renal tumor (Case 7). (a) Sagittal view showing partial agenesis of corpus callosum with absent rostrum and genum; corpus callosum fragment is located posterior to mammillary body–anterior commissure–corpus callosum line (black line). (b) Axial view showing reduction in parenchymal thickness of medial frontal lobes with secondary enlargement of interhemispheric space and frontal horns (arrow).

For fetuses affected by extensive autolysis, dissection and microscopic evaluation were unable to identify partial corpus callosum agenesis (Case 7) (Figure 1) and severe hydrocephaly (Case 8) which were seen on virtual autopsy. Statistically, these were considered to be false positives.

**Figure 2:**
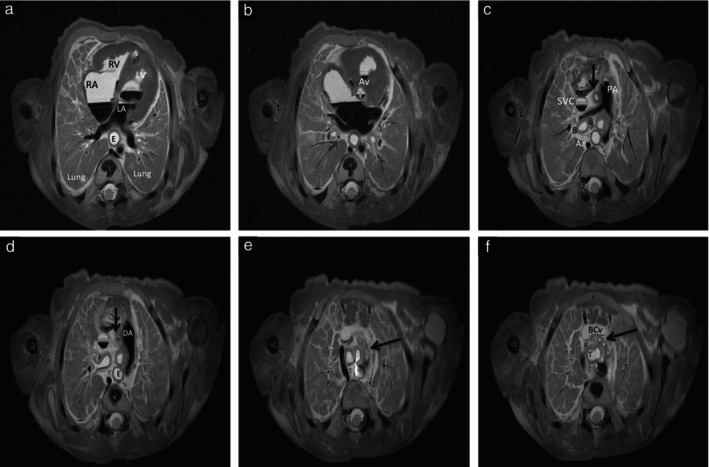
Fetal virtopsy using 7‐Tesla turbo spin‐echo high‐resolution T2‐weighted magnetic resonance imaging in axial plane in fetus at 18 weeks' gestation following termination of pregnancy for cardiac malformation (Case 6). (a) Left ventricle (LV) hypertrophy and reduction in cavity size. (b) Normal appearance of aortic valve (Av). (c) Preductal coarctation of the aorta (arrow). (d) Dilated appearance of ductus arteriosus (DA), probably compensatory. (e) Aortic arch with narrowed aspect (arrow). (f) Three branches (arrow) emerging from aortic arch. Az, azygos vein; B, bronchus; BCv, left brachiocephalic vein; E, esophagus; LA, left atrium; PA, pulmonary artery; RA, right atrium; RV, right ventricle; SVC, superior vena cava; T, trachea.

**Figure 3:**
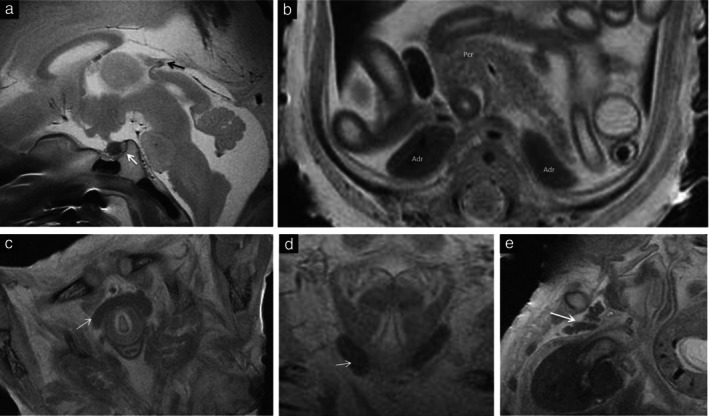
Fetal virtopsy using 7‐Tesla turbo spin‐echo high‐resolution T2‐weighted magnetic resonance imaging, depicting fetal endocrine glands in sagittal (a,e), axial (b,c) and coronal (d) views: (a) hypophysis (white arrow) and pineal gland (black arrow); (b) pancreas (Pcr) and adrenal glands (Adr); (c) thyroid gland (arrow); (d) parathyroid gland (arrow); (e) thymus (arrow).

Virtual autopsy did not identify intestinal malrotation which was confirmed at classical autopsy in five cases (Cases 17, 23, 25, 26 and 30), and missed three cases of membranous ventricular septal defect confirmed at classical autopsy using microscopy (Cases 2, 19 and 25). However, virtual autopsy performed very well, even for the examination of the great vessels, and identified difficult‐to‐detect anomalies such as preductal coarctation of the aorta in small fetuses weighing ≤ 300 g (Figure 2). One case of lung segmentation abnormality was not identified by virtual autopsy (Case 1), and virtual autopsy had one false‐negative result for the identification of cervical rachischisis (Case 18). The renal system was easy to depict on 7‐T MRI, including the bladder, kidneys, ureters and fetal endocrine glands (Figure 3). Details of the imaging and autopsy findings of the included cases are shown in Table S2.

### Diagnostic accuracy of virtopsy according to gestational age

To assess the concordance between 7‐T virtual autopsy and classical autopsy at different gestational ages, the fetuses were grouped into three gestational‐age intervals based on embryological development (Table 3). The degree of concordance between the two procedures was directly proportional to the gestational age and fetal weight. The sensitivity of virtual autopsy at 7 T to describe structural abnormalities increased from 87.50% in the 13–14‐week subgroup to 93.88% in the 17–19‐week subgroup.

**Table 3:** Diagnostic accuracy of postmortem magnetic resonance imaging at 7 Tesla *vs* conventional autopsy for different gestational‐age (GA) intervals

GA at demise	Weight (g)	Sensitivity	Specificity	PPV	NPV	Accuracy	Cohen's κ	Malformation frequency	*P* *
13–14 weeks (*n* = 9)	26.2 (18–42)	87.50 [71.01–96.49]	100 [93.73–100]	100 [100–100]	93.44 [85.07–97.27]	95.51 [88.89–98.76]	0.899	35.96 [26.05–46.82]	0.1336
15–16 weeks (*n* = 9)	112.7 (56–200)	93.75 [79.19–99.23]	96.00 [86.29–99.51]	93.75 [79.37–98.32]	96.00 [86.23–98.92]	95.12 [87.98–98.66]	0.897	39.02 [28.44–50.43]	0.6171
17–19 weeks (*n* = 12)	188.5 (68–300)	93.88 [83.13–98.72]	98.77 [93.31–99.97]	97.87 [86.75–99.69]	96.39 [89.91–98.76]	96.92 [92.31–99.16]	0.933	37.69 [9.35–46.61]	0.6171

Data are given as mean (range) or % [95% CI].

* McNemar test.

NPV, negative predictive value; PPV, positive predictive value.

## DISCUSSION

This study shows that fetal virtopsy using 7‐T MRI performed similarly to classical autopsy in second‐trimester fetuses following spontaneous pregnancy loss or termination of pregnancy. Moreover, the preparation and scanning protocols for virtual autopsy are simple, reliable and easily reproducible. Our findings can be used as evidence to validate the use of postmortem MRI at 7 T for fetal virtopsy in clinical practice. Using a two‐dimensional protocol with a clinically acceptable mean scanning time of approximately 62 min, virtual autopsy at 7 T achieved a diagnostic accuracy of 96%, a sensitivity of 92% and a specificity of 98% to detect structural abnormalities in fetuses with an average weight of 120 g.

As expected, non‐invasive virtual autopsy successfully identified all structural cerebral abnormalities, confirming its potential to replace the classical method for evaluation of the fetal brain. Evaluation of the cardiovascular system using postmortem MRI has been considered for many years deficient compared with the classical autopsy method and depends on the type of anomaly, gestational age and magnetic field^7^. Our study had only three discordant results in the cardiovascular segment; all three anomalies were identified on microscopic examination. However, it should be noted that this excellent result could also be explained by the nature of the anomalies described in the classical four‐chamber view. The specificity and diagnostic accuracy of virtual autopsy for cardiac anomalies should increase with extensive training of specialists in the fetal cord and improved communication between reference centers^15^. The lowest diagnostic accuracy of virtual autopsy using MRI at 7 T was found for analysis of the digestive system. Evaluation of the intestine is always an issue for postmortem imaging^15^, and increasing the magnetic field strength does not seem to improve the outcome, which emphasizes the need for new strategies to evaluate the intestine and overcome the current limitations of MRI. Similar to other studies, we found a decrease in diagnostic accuracy of fetal virtopsy in smaller fetuses with lower birth weights^7^, ^16^, ^17^.

A particular feature of our study was the use of 10% formaldehyde for up to 1 week and storage of fetuses at 4°C until scanning. Temperature is known to influence the quality of postmortem T2‐WI^18^, ^19^. The contrast of tissues rich in water (e.g. brain tissue) is higher at low temperatures; however, body temperature must be increased for soft tissues to obtain appropriate contrast^19^. Therefore, we started with scans of the nervous segment and continued with the thoracic and abdominal segments. Initially, formaldehyde was used to slow autolysis^20^. Later, after the formaldehyde had been removed, we observed that tissue contrast improved, especially in embryos^6^. However, for cerebral malformations, such as hydrocephaly, we recommend commencing scanning as soon as possible. The best images were obtained in cases with a short interval between delivery and scanning, probably because the cerebrospinal fluid maintained excellent contrast. However, the possibility of confirming anomalies by autopsy decreases with each day of autolysis. More studies are needed to measure precisely the temperature inside the scanning coil and determine its influence on the contrast obtained when scanning specific fetal segments.

To examine small fetal anatomy, 9.4‐T MRI or microfocus computed tomography (CT) is preferred^21^, ^22^. These methods have different advantages and disadvantages. Microfocus CT is more widely accepted because of its affordability and shorter scanning time. However, images obtained after microfocus CT staining have a lower volume compared with those obtained from non‐staining postmortem UHF‐MRI^1^, ^8^. MRI remains a valuable reference point because of its high resolution for soft tissue, and it does not require iodine staining. The preparation time required for MRI is less than that for CT, and, in contrast to iodine, formaldehyde for postmortem MRI does not cause tissue shrinkage^8^. In view of the COVID‐19 pandemic, virtual autopsy offers the advantage of limiting human contact while offering quality information for diagnosis; therefore, it has potential for postmortem evaluation in future infectious settings.

Virtual and classical autopsy had similar costs at the time of enrolment in this study, which was about 20% of the average monthly Romanian household income^23^. However, virtual autopsy offers a more cost‐effective solution, as it significantly reduces the time required compared with classical autopsy. From a clinical perspective, the most significant cost driver is the need for specialized personnel, as both investigation methods require more specialists in the field. Telemedicine may mitigate this issue, as the imaging approach not only enables a single specialist to interpret acquisitions from several centers, but also eliminates the need for their physical presence during the image‐acquisition process. Furthermore, for a complex case, the same image can be assessed by multiple specialists, facilitating a more comprehensive and accurate diagnosis. Unlike the classical autopsy approach, in which the dissection technique and the first examiner's experience directly influence the quality of the result, the imaging approach is more standardized and reproducible, with the potential for enhancement using artificial intelligence. This makes it a viable alternative, particularly in the face of the aforementioned challenges, and underscores its utility in medical research.

An important strength of this study is that we included consecutive cases, both normal and malformed, all of which were examined using the accepted gold‐standard method of classical autopsy. Therefore, our findings accurately reflect the diagnostic potential of 7‐T MRI for the postmortem evaluation of small fetuses. Moreover, the radiologist and embryologist were blinded to ultrasonographic, clinical and autopsy findings. By blinding the radiologist to the autopsy results, we could evaluate the diagnostic accuracy of fetal virtopsy and not our learning curve. In clinical practice, clinical and ultrasound data can only improve the results of postmortem imaging analysis.

The limitations of this study include the small number of enrolled cases. The cases were recruited from a single medical center, and only structural anomalies were considered, which may explain the high level of concordance with the gold standard.

In conclusion, fetal virtopsy using UHF‐MRI at 7 T is a feasible postmortem diagnostic tool to confirm structural anomalies in small fetuses weighing 17–364 g after fixation in 10% formaldehyde for up to 1 week. The diagnostic accuracy of postmortem virtual autopsy of malformed fetuses aged 13–19 weeks using 7‐T MRI is similar to that of the gold standard, classical autopsy, when analyzing the pulmonary, cardiovascular and renal systems, superior for analyzing the nervous system in small fetuses with pronounced autolysis, but inferior when evaluating the fetal intestines.

## Supporting information


**Table S1** Modified magnetic resonance imaging acquisition parameters on sagittal sections in 30 cases, according to fetal characteristics
**Table S2** Overview of virtual and classical autopsy findings in fetuses

## Data Availability

The data supporting the findings of this study are available in Figshare with the identifier https://figshare.com/s/fa280cc84cb34fc04ae4.

## References

[1] O'Keefe H , Shenfine R , Brown M , Beyer F , Rankin J . Are non‐invasive or minimally invasive autopsy techniques for detecting cause of death in prenates, neonates and infants accurate? A systematic review of diagnostic test accuracy. BMJ Open. 2023;13(1):e064774. 10.1136/bmjopen-2022-064774 PMC9827258 36609326

[2] Shelmerdine SC , Arthurs OJ , Gilpin I , et al. Is traditional perinatal autopsy needed after detailed fetal ultrasound and post‐mortem MRI? Prenat Diagn. 2019;39(9):818‐829. 30892705 10.1002/pd.5448

[3] Thali MJ , Jackowski C , Oesterhelweg L , Ross SG , Dirnhofer R . VIRTOPSY – the Swiss virtual autopsy approach. Leg Med (Tokyo). 2007;9(2):100‐104. 17275386 10.1016/j.legalmed.2006.11.011

[4] Lewis C , Hutchinson JC , Riddington M , et al. Minimally invasive autopsy for fetuses and children based on a combination of post‐mortem MRI and endoscopic examination: a feasibility study. Health Technol Assess. 2019;23(46):1‐104. 10.3310/hta23460 PMC6732714 31461397

[5] Sébille SB , Rolland AS , Welter ML , Bardinet E , Santin MD . Post mortem high resolution diffusion MRI for large specimen imaging at 11.7 T with 3D segmented echo‐planar imaging. J Neurosci Methods. 2019;1(311):222‐234. 10.1016/j.jneumeth.2018.10.010 30321565

[6] Staicu A , Albu C , Popa‐Stanila R , et al. Potential clinical benefits and limitations of fetal virtopsy using high‐field MRI at 7 Tesla versus stereomicroscopic autopsy to assess first trimester fetuses. Prenat Diagn. 2019;39(7):505‐518. 30980413 10.1002/pd.5457

[7] Votino C , Jani J , Verhoye M , et al. Postmortem examination of human fetal hearts at or below 20 weeks' gestation: a comparison of high‐field MRI at 9.4 T with lower‐field MRI magnets and stereomicroscopic autopsy. Ultrasound Obstet Gynecol. 2012;40(4):437‐444. 22605566 10.1002/uog.11191

[8] Dawood Y , Strijkers GJ , Limpens J , Oostra RJ , de Bakker BS . Novel imaging techniques to study postmortem human fetal anatomy: a systematic review on microfocus‐CT and ultra‐high‐field MRI. Eur Radiol. 2020;30(4):2280‐2292. 31834508 10.1007/s00330-019-06543-8 PMC7062658

[9] Kang X , Carlin A , Cannie MM , Sanchez TC , Jani JC . Fetal postmortem imaging: an overview of current techniques and future perspectives. Am J Obstet Gynecol. 2020;223(4):493‐515. 32376319 10.1016/j.ajog.2020.04.034

[10] O'Brien BC , Harris IB , Beckman TJ , Reed DA , Cook DA . Standards for reporting qualitative research: a synthesis of recommendations. Acad Med. 2014;89(9):1245‐1251. 24979285 10.1097/ACM.0000000000000388

[11] E‐Razavi F , Carles D , Bouvier R , Dauge MC . Pathologie Foetale et Placentaire Pratique. Sauramps Médical; 2008:536.

[12] Interactive Statistical Calculation Pages [Internet]. cited October 12, 2023. https://statpages.info/.

[13] Buderer NM . Statistical methodology: I. Incorporating the prevalence of disease into the sample size calculation for sensitivity and specificity. Acad Emerg Med. 1996;3(9):895‐900. 8870764 10.1111/j.1553-2712.1996.tb03538.x

[14] Leeflang MMG , Allerberger F . Sample size calculations for diagnostic studies. Clin Microbiol Infect. 2019;25(7):777‐778. 30986555 10.1016/j.cmi.2019.04.011

[15] Arthurs OJ , Thayyil S , Owens CM , et al. Diagnostic accuracy of post mortem MRI for abdominal abnormalities in foetuses and children. Eur J Radiol. 2015;84(3):474‐481. 25533719 10.1016/j.ejrad.2014.11.030

[16] Ulm B , Dovjak GO , Scharrer A , et al. Diagnostic quality of 3Tesla postmortem magnetic resonance imaging in fetuses with and without congenital heart disease. Am J Obstet Gynecol. 2021;225(2):189.e1‐189.e30. 10.1016/j.ajog.2021.02.030 33662361

[17] Taylor AM , Sebire NJ , Ashworth MT , et al. Postmortem cardiovascular magnetic resonance imaging in fetuses and children: a masked comparison study with conventional autopsy. Circulation. 2014;129(19):1937‐1944. 24647275 10.1161/CIRCULATIONAHA.113.005641

[18] Kobayashi T , Shiotani S , Kaga K , et al. Characteristic signal intensity changes on postmortem magnetic resonance imaging of the brain. Jpn J Radiol. 2010;28(1):8‐14. 20112087 10.1007/s11604-009-0373-9

[19] Berger C , Bauer M , Wittig H , Scheurer E , Lenz C . Post mortem brain temperature and its influence on quantitative MRI of the brain. Magma. 2022;35(3):375‐387. 34714448 10.1007/s10334-021-00971-8 PMC9188516

[20] Thavarajah R , Mudimbaimannar VK , Elizabeth J , Rao UK , Ranganathan K . Chemical and physical basics of routine formaldehyde fixation. J Oral Maxillofac Pathol. 2012;16(3):400‐405. 23248474 10.4103/0973-029X.102496 PMC3519217

[21] Shelmerdine SC , Arthurs OJ . Post‐mortem perinatal imaging: what is the evidence? Br J Radiol. 2023;96(1147):20211078. 35451852 10.1259/bjr.20211078 PMC10321257

[22] Docter D , Dawood Y , Jacobs K , et al. Microfocus computed tomography for fetal postmortem imaging: an overview. Pediatr Radiol. 2023;53(4):632‐639. 36169668 10.1007/s00247-022-05517-1 PMC10027643

[23] Institutul National de Statistica [Internet]. Accessed August 10, 2024. https://insse.ro/cms/sites/default/files/com_presa/com_pdf/cs01r17.pdf.

