# Supplementary material for: Whole‐body non‐forensic fetal virtopsy using postmortem magnetic resonance imaging at 7 Tesla vs classical autopsy
Source: Ultrasound Obstet Gynecol. 2024 Oct 7;64(5):661–8. doi: 10.1002/uog.29106 (PMC11579436; doi:10.1002/uog.29106)
Supplement: Supplementary file 1 — Table S1 Modified magnetic resonance imaging acquisition parameters on sagittal sections in 30 cases, according to fetal characteristics Table S2 Overview of virtual and classical autopsy findings in fetuses [file UOG-64-661-s001.docx]

**Table S1 Modified magnetic resonance imaging acquisition parameters on sagittal sections in 30 cases, according to fetal characteristics**

| **Case** | **Effective echo time (ms)** | **Repetition time (ms)** | **Slice thickness (mm)** | **Field of view (mm)** | **Matrix (data points)** | **Resolution (cm/pixel)** | **Number of slices** | **Scanning time (min)** |
| --- | --- | --- | --- | --- | --- | --- | --- | --- |
| 1 | 40.2 | 5252.1 | 0.8/1.3 | 4.85 | 384×384 | 0.0126/0.0994 | 38 | 42 |
| 2 | 41.4 | 5530.9 | 0.75/1 | 4 | 512×512 | 0.0078/0.0069 | 28 | 29.29 |
| 3 | 36 | 3916.5 | 0.5/0.75 | 3.73 | 384×384 | 0.0097/0.0079 | 33 | 15.39 |
| 4 | 36 | 6646.2 | 0.5/0.75 | 3.93 | 384×384 | 0.0102/0.0123 | 55 | 26 |
| 5 | 41.4 | 11168 | 0.5/0.75 | 6.65 | 512×512 | 0.0130/0.0123 | 70 | 126 |
| 6 | 41.4 | 5530.9 | 0.75/1 | 4 | 512×512 | 0.0078/0.0069 | 28 | 29 |
| 7 | 36 | 6527.6 | 0.5/0.75 | 6 | 384×384 | 0.0156/0.0131 | 52 | 26.35 |
| 8 | 36 | 8307.8 | 0.6/0.85 | 4.93 | 383×383 | 0.0128/0.0144 | 67 | 33.13 |
| 9 | 36 | 8764.9 | 0.6/0.85 | 5.73 | 384×384 | 0.0149/0.0144 | 57 | 27 |
| 10 | 36 | 5340.7 | 0.75/1 | 3.73 | 384×384 | 0.0098/0.0131 | 40 | 21 |
| 11 | 36 | 6646.2 | 0.5/0.75 | 2.73 | 384×384 | 0.0710/0.0112 | 56 | 26 |
| 12 | 36 | 5340.7 | 0.75/1 | 3.73 | 384×384 | 0.0098/0.0131 | 40 | 21 |
| 13 | 36 | 5696.8 | 0.6/0.85 | 4.75 | 384×384 | 0.0124/0.0125 | 48 | 22.47 |
| 14 | 36 | 5340.7 | 0.5/0.75 | 4 | 384×384 | 0.0104/0.0060 | 40 | 21 |
| 15 | 36 | 4747.3 | 0.5/0.75 | 4.1 | 384×384 | 0.0107/0.0104 | 38 | 18 |
| 16 | 36 | 7714.4 | 0.5/0.75 | 4.9 | 384×384 | 0.0154/0.0149 | 59 | 30 |
| 17 | 36 | 5934.1 | 0.5/0.75 | 3.73 | 384×384 | 0.0097/0.0104 | 44 | 23 |
| 18 | 36 | 5934.1 | 0.5/0.75 | 4.73 | 384×384 | 0.0123/0.0121 | 48 | 23 |
| 19 | 36 | 5340.7 | 0.5×0.75 | 4 | 384×384 | 0.0104/0.0060 | 40 | 21 |
| 20 | 36 | 6527.6 | 0.5/0.75 | 6 | 384×384 | 0.0156/0.0131 | 52 | 26 |
| 21 | 36 | 6646.2 | 0.5/0.75 | 2.73 | 384×384 | 0.0710/0.0112 | 56 | 26 |
| 22 | 36 | 9494.6 | 0.5/0.75 | 5.95 | 384×384 | 0.0155/0.0151 | 70 | 37 |
| 23 | 36 | 5340.7 | 0.5/0.75 | 4 | 384×384 | 0.0104/0.0072 | 45 | 21 |
| 24 | 36 | 3441.8 | 0.5/0.75 | 4 | 384×384 | 0.0104/0.0092 | 29 | 13.37 |
| 25 | 36 | 5104.4 | 0.6/0.85 | 4 | 384×384 | 0.0104/0.0104 | 38 | 20 |
| 26 | 36 | 6883.6 | 0.5/0.75 | 5.93 | 384×384 | 0.0154/0.0115 | 55 | 27.32 |
| 27 | 36 | 4628.6 | 0.5/0.75 | 3.73 | 384×384 | 0.0970/0.0092 | 39 | 18.30 |
| 28 | 36 | 62909.2 | 0.5/0.75 | 3.13 | 384×384 | 0.0820/0.0104 | 53 | 26 |
| 29 | 36 | 9494 | 0.5/0.75 | 4.5 | 384×384 | 0.0117/0.0117 | 80 | 37.58 |
| 30 | 36 | 6883.6 | 0.5/0.75 | 5.93 | 384×384 | 0.0154/0.0115 | 55 | 27.32 |

**Table S2** Overview of virtual and classical autopsy findings in fetuses

| **No. of case** | **Gestational week** | **Weight (g)** | **Karyotype/other genetic test** | **Postmortem findings** | | **Disagreement** | **Observation** |
| --- | --- | --- | --- | --- | --- | --- | --- |
|  |  |  |  | **Virtual autopsy** | **Classical autopsy** |  |  |
| 1. | 15 | 89 | Not tested | Anencephaly, craniospinal rachischisis. Exophthalmia, absent neurohypophysis | Anencephaly, craniospinal rachischisis, exolphalmy, absent neurohypophysis, incomplete lobar segmentation of the right lung | Incomplete segmentation of the right lung (CA) |  |
| 2. | 13 | 20 | Not tested | Retrognathia | Retrognathia, membranous VSD | Membranous VSD (microscopy) (CA) |  |
| 3. | 16 | 120 | Trisomy 18 | Dilated cerebral ventricles, right lung with two lobes, nonseptated left lung, bilateral duplex collecting system, horseshoe kidney, polycystic renal dysplasia | Dilated cerebral ventricles, right lung with two lobes, nonseptated left lung, bilateral duplex collecting system, horseshoe kidney, polycystic renal dysplasia | None |  |
| 4. | 14 | 25 | Not tested | Female fetus without structural anomalies | Female fetus without structural anomalies | None | Maternal pathology indication for TOP |
| 5. | 19 | 300 | Normal | Preductal coarctation of the aorta with tubular hypoplasia of the aortic arch | Preductal coarctation of the aorta with tubular hypoplasia of the aortic arch | None |  |
| 6. | 18 | 250 | Normal | Preductal coarctation of the aorta | Preductal coarctation of the aorta | None |  |
| 7. | 16 | 200 | Normal | pACC, uretero-renal malformation (unilateral ectopic opening of the ureter and left cystic renal tumor) | Left cystic nephroma | pACC (VA) |  |
| 8. | 17 | 120 | Not tested | Hydrocephaly, possible aortic coarctation, hypointense hepatic masses | Preductal aortic stenosis, extensive autolysis of the cerebral tissue | Hydrocephaly (VA) | Extensive autolysis |
| 9. | 16 | 167 | Not tested | None | None | None | Spontaneous abortion |
| 10. | 17 | 169 | Duchenne muscular dystrophy | None | None | None |  |
| 11. | 18 | 289 | Trisomy 21 | None | None | None |  |
| 12. | 17 | 150 | Not tested | Occipital meningoencephalocele, ventriculomegaly | Occipital meningoencephalocele, ventriculomegaly | None |  |
| 13. | 19 | 364 | Normal | Left ventricle hypoplasia, atrioventricular canal, aortic stenosis, polysplenia | Three-chambered heart, left ventricle hypoplasia, aortic stenosis, VSD, dilated arterial canal, possible left hypoplastic ventricle syndrome with craniofacial dysmorphism and accessory spleens | None |  |
| 14. | 18 | 68 | 69XXY | Intraventricular hemorrhage, atrial septal defect, left cheilo-gnatho-palatoschisis, cystic hygroma, hypotrophic fetus | Intraventricular hemorrhage, atrial septal defect, left cheilo-gnatho-palatoschisis, cystic hygroma, hypotrophic fetus | None |  |
| 15. | 18 | 192 | Trisomy 21 | Preductal coarctation of the aorta, aberrant right subclavian artery (Lusoria artery), bilateral hydronephrosis, facial dysmorphism | Preductal coarctation of the aorta, aberrant right subclavian artery (Lusoria artery), lung hypoplasia, bilateral hydronephrosis, nuchal edema, microretrognathia, low-onset ears | None |  |
| 16. | 13 | 42 | Trisomy 13 | Semilobar holoprosencephaly, Dandy–Walker anomaly, corpus callosum agenesis, cheiloschisis, hypotelorism | Semilobar holoprosencephaly, Dandy–Walker anomaly, corpus callosum agenesis, cheiloschisis, nuchal edema, low-onset ears | None |  |
| 17. | 16 | 100 | Normal | Laparoschisis, aortic stenosis, secondary left ventricular hypertrophy, hypertelorism, low-onset ears | Laparoschisis, aortic stenosis, secondary left ventricular hypertrophy, intestinal rotation disorder, hypertelorism, low-inserted ears, nuchal edema | Intestinal malrotation (CA) |  |
| 18. | 17 | 98 | Normal | Anencephaly, cheilopalatoschisis, left lung with one lobe, right lung with two lobes | Anencephaly, craniospinal rachischisis, improper lung lobation, cheilopalatoschisis | Cervical rachischisis (CA) |  |
| 19. | 15 | 54 | Not tested | Microretrognathia, low-inserted ears, congenital clubfoot, posterior fossa anomaly with cerebellar hypoplasia | Microretrognathia, low-inserted ears, congenital clubfoot, membranous defect of the interventricular septum, posterior fossa anomaly with cerebellar hypoplasia | Membranous VSD (CA at microscopy) |  |
| 20. | 16 | 80 | Trisomy 18 | Atrioventricular canal, omphalocele with hepatic herniation, lumbar spina bifida with meningocele, microretrognathia, cystic hygroma | Omphalocele, rachischisis, complex cardiac malformation type atrioventricular canal type I Rastelli, microretrognathia, hypertrophy | None | Highly macerated aspect of the cerebral matter |
| 21. | 19 | 215 | Not tested | Dandy–Walker malformation, cleft palate | Cerebellar dysgenesis, facial dysmorphism, cleft palate | None |  |
| 22. | 13 | 22 | Not tested | Right subclavian artery emerging separately from the aortic arch | None | Right subclavian artery emerging separately from the aortic arch (VA) |  |
| 23. | 13 | 21 | Not tested | Posttraumatic changes at the base of the skull, bifid cardiac apex, bilateral hydronephrosis | Left suprarenal nephroblastoma; poorly differentiated infiltrate in the retroperitoneal space metastasizing in the homolateral kidney, mediastinum, and placenta; bifid cardiac apex; hydronephrosis; intestinal malrotation | Intestinal malrotation (CA) |  |
| 24. | 16 | 55 | Trisomy 18 | Cardiomegaly with complex cardiac malformation comprising: right ventricle with double exit pathway, aortic valve stenosis, pulmonary artery stenosis, mitral stenosis, defect of the interatrial septum, aortic arch abnormality; pulmonary hypoplasia; microretrognathia; hypertelorism; congenital single kidney; left renal agenesis; polymicrogyria; corpus callosum agenesis | Corpus callosum agenesis, cardiomegaly with complex cardiac malformation comprising: right ventricle with double exit pathway, aortic valve stenosis, pulmonary artery stenosis, mitral stenosis, defect of the interatrial septum, endocardial fibroelastosis, aortic arch abnormality; pulmonary hypoplasia; nuchal edema; minor microretrognathia; hypertelorism; low-inserted ears; intestinal malrotation; congenital single kidney; left renal agenesis; thymic hypoplasia |  |  |
| 25. | 13 | 23 | 69XXY | Craniofacial dysmorphism, dwarfism, vermis hypoplasia, posterior fossa enlargement, hydrocephaly, pulmonary artery stenosis, omphalocele, bilateral renal hypoplasia | Craniofacial dysmorphism, thanatophoric dwarfism, vermis hypoplasia, posterior fossa enlargement, hydrocephaly, pulmonary artery stenosis type with interventricular septal defect, omphalocele, intestinal malrotation, bilateral renal hypoplasia, bone prominence of 0.5/0.5 cm in the sacral region | Intestinal malrotation(CA)  VSD (CA) |  |
| 26. | 13 | 17 | Not tested | Anencephaly, cervico-thoraco-lumbar craniosahischisis with spine retroflexion, cervical and thoracic spine kyphosis, transposition of large vessels, aortic arch abnormality, postductal aortic coarctation, left kidney hydronephrosis | Anencephaly, cervico-thoraco-lumbar craniosahischisis with spine retroflexion, cervical and thoracic spine kyphosis, transposition of large vessels, aortic arch abnormality, postductal aortic coarctation, left kidney hydronephrosis, intestinal malrotation | Intestinal malrotation (CA) |  |
| 27. | 17 | 101 | Not tested | Pentalogy of Cantrell with pulmonary extrophy, craniorachischisis, hypophyseal agenesis | Pentalogy of Cantrell with pulmonary extrophy, craniorachischisis, right-sided aortic arch with aberrant brachiocephalic artery | Right-sided aortic arch with aberrant brachiocephalic artery (CA) |  |
| 28. | 15 | 90 | Not tested | None | None | None | Spontaneous abortion |
| 29. | 13 | 27 | Normal | Prune-belly syndrome | Prune-belly syndrome |  |  |
| 30. | 13 | 33 | Normal | Left ventricular hypoplasia, aortic valve stenosis, cystic hygroma | Left ventricular hypoplasia, aortic valve stenosis, intestinal malrotation, cystic hygroma | Intestinal malrotation (CA) |  |

CA, classical autopsy; pACC, partial agenesis of corpus callosum; TOP, termination of pregnancy; VA, virtual autopsy; VSD, ventricular septal defect.
